# A Comparison of Computer-Aided Diagnosis Schemes Optimized Using Radiomics and Deep Transfer Learning Methods

**DOI:** 10.3390/bioengineering9060256

**Published:** 2022-06-15

**Authors:** Gopichandh Danala, Sai Kiran Maryada, Warid Islam, Rowzat Faiz, Meredith Jones, Yuchen Qiu, Bin Zheng

**Affiliations:** 1 School of Electrical and Computer Engineering, University of Oklahoma, Norman, OK 73019, USA; warid9666@ou.edu (W.I.); rowzat@ou.edu (R.F.); qiuyuchen@ou.edu (Y.Q.); 2 School of Computer Science, University of Oklahoma, Norman, OK 73019, USA; sai.maryada@ou.edu; 3 Stephenson School of Biomedical Engineering, University of Oklahoma, Norman, OK 73019, USA; meredith.jones@ou.edu

**Keywords:** computer-aided diagnosis (CAD) schemes, radiomics, deep transfer learning, breast lesion classification, assessment of CAD performance

## Abstract

Objective: Radiomics and deep transfer learning are two popular technologies used to develop computer-aided detection and diagnosis (CAD) schemes of medical images. This study aims to investigate and to compare the advantages and the potential limitations of applying these two technologies in developing CAD schemes. Methods: A relatively large and diverse retrospective dataset including 3000 digital mammograms was assembled in which 1496 images depicted malignant lesions and 1504 images depicted benign lesions. Two CAD schemes were developed to classify breast lesions. The first scheme was developed using four steps namely, applying an adaptive multi-layer topographic region growing algorithm to segment lesions, computing initial radiomics features, applying a principal component algorithm to generate an optimal feature vector, and building a support vector machine classifier. The second CAD scheme was built based on a pre-trained residual net architecture (ResNet50) as a transfer learning model to classify breast lesions. Both CAD schemes were trained and tested using a 10-fold cross-validation method. Several score fusion methods were also investigated to classify breast lesions. CAD performances were evaluated and compared by the areas under the ROC curve (AUC). Results: The ResNet50 model-based CAD scheme yielded AUC = 0.85 ± 0.02, which was significantly higher than the radiomics feature-based CAD scheme with AUC = 0.77 ± 0.02 (*p* < 0.01). Additionally, the fusion of classification scores generated by the two CAD schemes did not further improve classification performance. Conclusion: This study demonstrates that using deep transfer learning is more efficient to develop CAD schemes and it enables a higher lesion classification performance than CAD schemes developed using radiomics-based technology.

## 1. Introduction

Medical images are routinely used in clinical practice to detect and to diagnose diseases including cancer. However, reading and interpreting medical images is often a difficult and time-consuming task for radiologists, which does not only reduce diagnostic accuracy but also generates large intra- and inter-reader variability [1]. For example, full-field digital mammography (FFDM) is the most popular imaging modality used in the general population-based breast cancer screening in order to detect breast cancer at an early stage. However, due to two-dimensional projection imaging, FFDM has a relatively lower cancer detection sensitivity and specificity [2], particularly to detect and to classify subtle breast lesions in women of a younger age and/or having dense breast tissue [3]. Additionally, the higher false-positive recall and biopsy rates do not only increase healthcare cost but also add anxiety to patients with potentially long-term psychosocial consequences [4].

Thus, in order to address and to overcome this challenge to help radiologists to more accurately and efficiently read and diagnose medical images (i.e., FFDM images), developing computer-aided detection and diagnosis (CAD) schemes of medical images has been attracting broad research interest in the last several decades [5,6]. For CAD of mammograms, the computer-aided detection (CADe) schemes of suspicious lesion detection have been implemented in many medical centers or hospitals to assist radiologists in reading screening mammograms [7]. However, although great research effort has been made to develop computer-aided diagnosis (CADx) schemes of lesion classification [8,9], no CADx schemes have been approved and accepted in clinical practice. In this study, we focus on developing computer-aided diagnosis schemes of mammograms in order to help improve accuracy of lesion classification. In the following sections of this paper, CAD represents computer-aided diagnosis. If successful, applying CAD schemes to assist radiologists in classifying between malignant and benign breast lesions will have high clinical impact to help to significantly reduce false-positive recalls and unnecessary biopsies in future clinical practice.

In recent years, most CAD schemes are developed using either radiomics image features or deep learning models. When using radiomics concept, CAD schemes initially extract and compute large number of handcrafted features (i.e., >1000) in order to detect the underlying phenomenon of suspicious breast lesions [10]. These radiomic features can be obtained from a wide range of characteristics, covering lesion morphology, density heterogeneity, texture patterns, and other frequency domain features. The previous studies have demonstrated the feasibility to identify differently optimal feature vectors that may highly associate with lesion type (i.e., malignant vs. benign) [11], grade [12] and/or prognosis [13]. However, using a radiomics approach often creates a challenge for how to accurately segment lesions from the images. The accuracy or scientific rigor of the computed radiomics features often depends on the accuracy of lesion segmentation. Lesion segmentation errors may have a negative impact in the final performance of CAD schemes.

When applying deep learning technology, CAD schemes automatically extract and compute image features from the existing deep learning models using the transfer learning concept [14]. In this approach, a deep learning model pre-trained using a large database of non-medical images is selected. Then, a small set of medical images are used to finetune the model and to extract the automated features for the specific application tasks. In addition, in this approach, the image features are typically computed from the fixed regions of interest (ROIs) or image patches without lesion segmentation. Many previous studies have demonstrated the feasibility of developing CAD schemes using automated features directly extracted by deep transfer learning [15,16]. However, the physicians (i.e., radiologists) often do not have a higher confidence to accept such “a black box” type of image-in and prediction-out scheme as a decision-making support tool [17]. Thus, how to provide more convincing scientific data or evidence to increase the confidence of physicians to accept or to consider deep learning model generated classification results is an important research task.

Since in previous studies, CAD schemes were separately developed using either handcrafted radiomics features or deep transfer learning model generated automated features using different and relatively small image datasets, it is difficult to compare the performance of CAD schemes developed using these two types of image features. As a result, the advantages and/or potential limitations of CAD schemes trained using the radiomics and automated features or methods have not been well investigated to date. In order to address this issue, we conducted a new study to explore the association between the traditional radiomics feature-based CADs and a deep transfer learning model-based CAD scheme in classifying between malignant and benign breast lesions using a relatively large and diverse image dataset, as well as the same 10-fold cross-validation method. Additionally, we also investigated whether fusion of classification scores generated by these two types of CAD schemes can further improve CAD performance in breast lesion classification.

## 2. Materials and Methods

### 2.1. Image Dataset

In our medical imaging research laboratory, we previously assembled a large and diverse de-identified retrospective database of full-field digital mammography (FFDM) images with multiple year screenings. All FFDM images were acquired using Hologic Selenia (Hologic Inc., Bedford, MA, USA) digital mammography machines, which have a fixed pixel size of 70 μm. The detailed patients’ demographic information, breast density distribution, and other image characteristics were reported in our previous studies [9,18]. In this study, we selected 3000 FFDM images from this existing database to assemble a specific dataset for this study. Each image in this dataset depicts a detected soft tissue mass lesion with biopsy verified clinical diagnostic result. Table 1 shows the distribution of the lesions, depicting craniocaudal (CC) and mediolateral oblique (MLO) views of left and right FFDM images. In summary, this dataset includes 1496 images that depict malignant lesions and 1504 images that depict benign lesions.

The center location of each suspicious lesion was previously marked by the radiologist. Since we focus only on the classification of soft tissue mass lesions in this study, all original FFDM images were first subsampled using a pixel averaging method with a kernel of 5 × 5 pixels, which increased the image pixel size to 0.35 mm. Then, using each marked lesion center as a reference, we extracted a region of interest (ROI) or patch that had a pixel size of 150 × 150 to cover all mass lesions in the dataset. The lesion center and the extracted ROI center overlapped. If part of the ROI was beyond the boundary of the original FFDM image (i.e., a small lesion that is detected close to the edge of the image), a zero-pad correction method was applied. The examples of ROIs with zero-pad correction will be demonstrated in the sample ROIs in the Results section of the paper. The same sized ROI or patch has been affectively used in our previous CAD studies (i.e., [8,9]).

**Figure 1:**
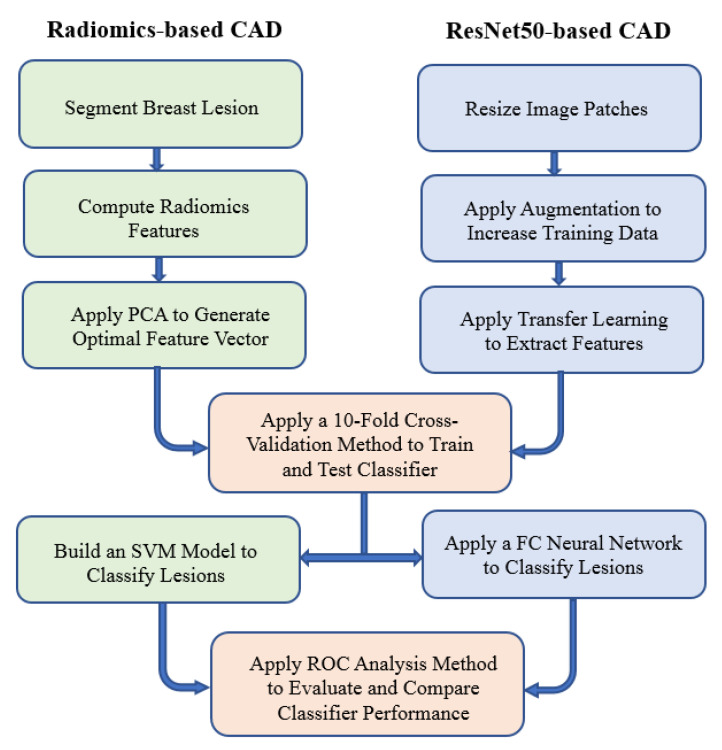
Illustration of each step to build two CAD schemes and to evaluate their performance in breast lesion classification.

Based on these extracted image ROIs, we built two CAD schemes, including a traditional CAD scheme implemented with a conventional machine learning classifier that was optimized using radiomics features and an automated CAD scheme implemented with a deep transfer learning model (ResNet50). Figure 1 illustrates the steps to build these two CAD schemes and to evaluate their performance to classify breast lesions. The detailed information of each image processing and analysis step is described in the following three subsections.

### 2.2. A CAD Scheme Using Radiomics Features

As shown in Figure 1, developing the radiomics feature based CAD scheme includes the following steps. First, we applied an adaptive multi-layer topographic region growing algorithm to segment lesion depicting in each ROI. Specifically, based on change of local lesion contrast in different topographic layers (j), adaptive region growing thresholds ($T_{j},\;j=1,\cdots n$) were computed using Equation (1) for the first growth layer and Equation (2) for the sequential layers.

(1) $$T_{1}=I_{seed}+\alpha I_{seed},\;\;\alpha =0.1$$

(2) $$T_{j}=T_{j-1}+\beta C_{j-1},\;\;\beta =0.5\;\;\;j=2,\ldots n$$

where $I_{seed}$ is the pixel value of the marked lesion center (growth seed), α and β are two pre-determined coefficients, $C_{j-1}$ is the region contrast at previous topographic layer (j−1), which is computed by a difference between the average pixel value of the lesion boundary contour and the internal lesion region in this layer.

Lesion segmentation was performed layer-by-layer until the growing resulted in the new layer violating one of two predetermined thresholds including (1) the ratio of lesion region size ($S_{j}$) increase and (2) the ratio of lesion circularity ($V_{j}$) reduction as defined and computed using Equations (3) and (4).

(3) $$G_{size-growth-ratio}=\frac{S_{j}-S_{j-1}}{S_{j-1}}>2.0$$

(4) $$V_{circularity-reduction-ratio}=\frac{\left|V_{j-1}-V_{j}\right|}{V_{j-1}}>0.5$$

These two growth termination thresholds prevent leakage of lesion growth to the surrounding background tissue region. Thus, if one of the above two thresholds was violated, the multi-layer topographic region growing stopped and the previous layer (j−1) was used to represent the final lesion regions segmented by the CAD scheme. This lesion segmentation algorithm has been tested in our previous studies (i.e., [18]). After applying this automated lesion segmentation algorithm, we also visually examined lesion segmentation results and manually corrected the possible segmentation errors (if any). Thus, we can reduce the errors or variations in computing lesion-associated radiomics features.

Second, after lesion segmentation, the CAD scheme initially computes a total of 235 traditional handcrafted image features that cover a variety of radiomic information representing lesion characteristics such as morphology, density heterogeneity, boundary contrast, texture patterns, and wavelet-based frequency domain features. These lesion-specific features explore and represent local patterns such as lesion shape, boundary spiculation, and density distribution within and around the boundary region of the lesion. The details of computing these radiomics features have been reported in our previous studies [19,20].

Third, many initially computed radiomics features can be highly redundant or irrelevant to lesion classification. Thus, we applied a standard principal component analysis (PCA) algorithm to process this initial feature pool of 235 features. The PCA was set to generate a new principal component feature vector with a variance rate of 95%, which has been proven as quite effective in reducing feature dimensionality and redundancy [20]. As a result, the PCA-generated optimal feature vector has a significantly smaller number of features, which can reduce feature redundancy and overfitting risk to train and to build a machine learning (ML) model to classify between malignant and benign lesions.

Fourth, although many different types of ML models have been investigated and applied in CAD schemes of medical images, we selected a support vector machine (SVM) as an ML model in this study because an SVM model uses a constructive ML process based on statistical learning theory to classify feature vectors into two classes of images (i.e., the images depicting malignant and benign lesions). By comparing with many other ML models, SVM has been approved with a minimal generalization error or a higher robustness [21], which makes SVM an optimal choice in medical image application with a relatively small image dataset. Thus, based on our previous experience [22], we selected a polynomial kernel to build the proposed SVM model in this study. A 10-fold cross-validation method was applied to train and to test this SVM classification model.

### 2.3. A CAD Scheme Using Deep Transfer Learning Model

The second CAD scheme used a deep learning architecture that was finetuned to extract automated image features. In recent years, many different deep learning models including AlexNet, VGG, DenseNet, Inception, and ResNet have been investigated as transfer learning models used in CAD schemes of image or lesion classification. Previous studies have compared the performance of applying different deep learning models in CAD schemes of medical images. For example, one recent study compared 32 deep learning models to detect and to classify different lung diseases. Among them, ResNet50 yields the highest classification accuracy [23]. Another study compared VGG-16, VGG-19, and ResNet50 and concluded that ResNet 50 was the best architecture framework for image classification task with the highest accuracy and efficiency to train [24]. Thus, in this study we selected the popular image classification architecture of residual net architecture (ResNet50) to build a deep transfer learning model used in our CAD scheme. The detailed architecture of ResNet50 has been previously described in reference [25]. In the original ResNet50, all network connection weights are pre-trained using a large color ImageNet dataset (with 3 RGB channels) to recognize or to classify 1000 different object classes.

In our CAD scheme, the original architecture of ResNet50 remained unchanged until the last fully connection (FC) feed-forward neural network, which was remodeled to classify only two classes namely, two classes of malignant and benign lesions. The following steps were applied to finetune the ResNet50 model to acquire transfer learning features and to train the lesion classifier. First, in order to use the ResNet50 model to extract automated features relevant to lesion characteristics depicted on mammograms, we applied several image preprocessing methods, which included (1) the originally extracted image ROI or patches of size 150 × 150 resized to the required size of 224 × 224 pixels using a bilinear interpolation algorithm, (2) the same grayscale FFDM image patch repeatedly input to the three channels of the ResNet50 model, and (3) a minimal augmentation step (involving random centered crop, random horizontal, and random vertical flip with *p* = 0.5) was added to introduce a slight variation of a sample image for different epochs (ROIs) during the training phase.

Next, due to the nature of medical images, a simple feature extractor type training involves the freezing of all unchanged layers and updates only the weights and biases of the modified last fully connection (FC) layer, which often does not yield satisfactory results. Thus, in this study, we finetune and optimize the weights of all layers of the ResNet50 model during the training. Specifically, given the limitation of our dataset size relative to other computer vision field, we maximize the training and consider the time required for this network-tuning. Specifically, we used a 10-fold cross-validation (CV) method. During each fold, the data was split randomly into training (90%) and testing (10%) without data repetition, and each sample case was used only once in the test phase. We investigated various batch sizes (i.e., 4, 8, 16, etc.) and observed that a batch size of four worked well for our analysis. Additionally, we selected an Adam optimizer with an initial learning rate of $10^{-4}$ at the beginning of each cross-validation fold. We updated the learning rate scheduler with an exponential decay function using a gamma value of 0.4 after each epoch. After each epoch, the network was evaluated to monitor training and validation loss during the training process, thereby deciding the stopping criterion. We noticed that by 10 epochs, the network was saturated, and any further training resulted in overfitting. Thus, we only trained the network for 10 epochs during each cross-validation fold. After model finetuning and training, images in the testing fold were then processed by the model. The last FC feed-forward neural network of the modified ResNet50 model generated a classification score of each testing image, which predicted a likelihood of the testing image depicting a malignant lesion.

### 2.4. Performance Evaluation and Comparison

After applying the 10-fold cross-validation method to train and to test the classifiers of two CAD schemes, each image in the dataset had two classification scores representing the probability or likelihood (from 0 to 1) of the image depicting a malignant breast lesion. We define the support vector machine (SVM) classifier used in a radiomics feature-based CAD scheme and a neural network (NN) used in the last fully-connected (FC) layer of ResNet50-based CAD scheme as Model-I and Model-II, with the classification scores as $S_{1}$ and $S_{2}$, respectively. In addition, we also test four fusion methods to build new models (Model-III) that combine two classification scores ($S_{1}$ and $S_{2}$). In model-III.1, $S_{1}$ and $S_{2}$ are used as two new features to build another SVM classifier. In model-III.2 to model-III.4, the following three simple score fusion methods are applied.

(1) Model-III.2, $S_{3.2}=W_{1}\times S_{1}+W_{2}\times S_{2}$. In this study, $W_{1}=0.5$ representing that the average score is used as the final classification score.
(2) Model-III.3, $S_{3.3}=\min\left(S_{1},\;S_{2}\right).$ The minimum score between Model-I and Model-II is used as the final classification score.
(3) Model-III.4, $S_{3.4}=\max\left(S_{1},\;S_{2}\right).$ The maximum score between Model-I and Model-II is used as the final classification score.

The similar CAD score fusion methods have been tested and applied in our previous studies, aiming to improve the lesion detection or the classification performance of CAD schemes [26,27].

Next, to evaluate and to compare the performance of each ML model, we applied the following two statistical data analysis steps. First, we used a receiver operating characteristic (ROC) type data analysis method. In order to reduce the potential bias of directly using raw scoring data to generate an unsmoothed ROC curve and to compute the area under the ROC curve (AUC), we used a maximum likelihood-based ROC curve fitting program (ROCKIT, http://metz-roc.uchicago.edu/MetzROC/software accessed on 8 May 2022) to generate a smoothed ROC curve. The corresponding AUC value along with the standard deviation (STD) was computed and used as an index to evaluate the performance of a CAD model to classify between malignant and benign breast lesions. The significant differences (*p*-values) between AUC values were also computed for comparing the classification performance of different models. Second, after applying an operation threshold on the model-generated classification scores (T = 0.5) to divide all testing cases into two classes (namely, score ≤ 0.5 represents a benign lesion and score > 0.5 represents a malignant lesion), we also computed and compared the overall classification accuracy (ACC) of different classification models as computed using Equation (5):

(5) $$\mathrm{ACC}=\frac{TM+TB}{All\;Images}$$

where TM and TB represent the numbers of correctly classified images depicting with malignant and benign lesions, respectively. All Images include the total number of images in the dataset. Both AUC and ACC along with the standard deviation (STD) are tabulated for comparison.

## 3. Results

**Figure 2:**
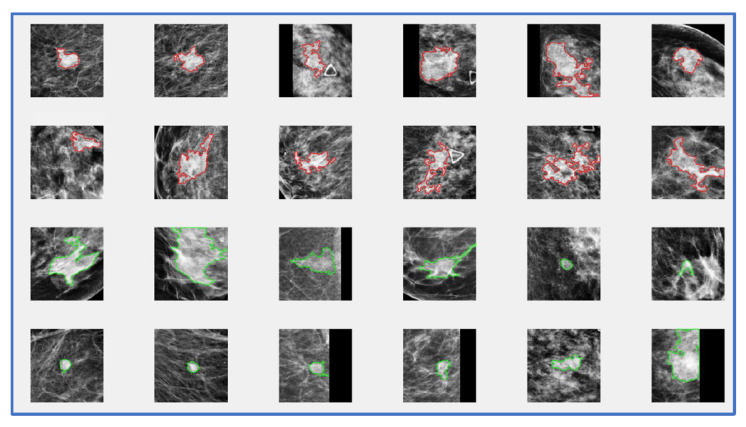
Illustration of sample image patches with lesion boundary contour segmentation overlay (in which Red and Green color marked boundary contours represent malignant and benign lesions, respectively).

Figure 2 shows 24 sample images included in our dataset with an overlay of lesion boundary segmentation results. The images with a segmentation overlay marked in a red or a green color represent malignant or benign lesions, respectively. The figure also shows that in seven images, zero paddings (black strips) are performed because these seven lesions are located near the edge or the corner inside the original image. From the density distribution of these lesions, we can observe both solid and diffused lesions. It is often challenging to segment the diffused or hidden lesions. The computed features and analysis results may not accurately represent the underlying lesion image marker. Despite such a challenge, our study results show that the lesion segmentation results are in general satisfactory and only a small subset (<5%) of images need a minor manual correction of CAD-segmented lesion boundary.

Table 2 summarizes and compares the lesion classification performance of six models including (1) AUC values and standard deviation (STD) computed from ROC curves and (2) overall classification accuracy (ACC) and STD after applying an operation threshold (T = 0.5) to the model-generated classification scores. In Model-I, PCA generates an optimal feature vector with 50 features, which is significantly reduced from the original 235 radiomics features in the initial feature pool. However, the AUC value of Model-I trained using a PCA-generated feature vector is 0.77 ± 0.02, which is significantly lower than the AUC value of 0.85 ± 0.02 generated by Model-II optimized using a deep transfer learning (ResNet50) model (*p* < 0.01). The four Model-III, which test four different fusion methods to combine classification scores generated by Model-I and Model-II, yield very comparable AUC values and no statistically significant differences are detected among these AUC values.

**Figure 3:**
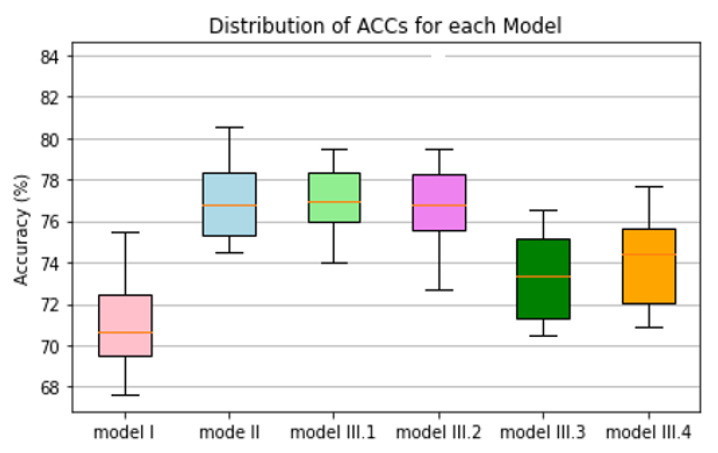
Illustration of six bar graphs representing distribution of overall accuracy of applying six models to classify between malignant and benign breast lesions.

After applying the operation threshold to divide images into two classes depicting malignant and benign lesions, the overall classification accuracy (ACC) of Model-II was also significantly higher than Model-I (as shown in Table 2). Additionally, Figure 3 shows the trend of bar patterns that represent the average ACC values and their overall distribution ranges among six models in which Model-III.1 that uses a new SVM model fusing with two classification scores generated by Model-I and Model-II yields the highest ACC = 77.42% ± 2.47%. However, it is not a statistically significant difference from ACC = 77.31% ± 2.65% generated by Model II (*p* = 0.87).

## 4. Discussion

Although many CAD schemes aiming to classify between malignant and benign breast lesions have been developed using different image processing algorithms and machine learning models, the reported classification performances vary greatly due to the use of different and smaller image datasets (i.e., AUCs ranging from 0.70 to 0.87 using datasets with 38 to 1200 images [28] or AUC = 0.76 ± 0.04 using a state-of-the-art VGG16 transfer learning model and an image dataset of 1535 images [29]). Thus, objectively comparing different CAD schemes and discussing their advantages or limitations is difficult. In this study, we investigate and systematically compare the performance of two CAD schemes that are developed using a popular conventional SVM model trained by a PCA-generated optimal radiomics feature vector and a deep transfer learning framework (ResNet50) to classify between malignant and benign breast lesions. Both CAD schemes are trained and tested using a 10-fold cross-validation method with a much larger image dataset involving 3000 lesion regions as compared to most previous studies (i.e., reviewed in [28]). Thus, this unique study generates several new and interesting observations, which may be useful to guide future CAD research to develop new CAD schemes with an improved classification accuracy and high scientific rigor or robustness.

First, radiomics and deep learning are two new concepts or advanced technologies widely adopted in the current CAD field. Although which approach can yield a significantly higher performance is still debatable, particularly when using small training image datasets. This study demonstrated that a CAD scheme optimized using a deep transfer learning model (i.e., ResNet50) yields a significantly higher performance for classifying breast lesions than using the scheme optimized using radiomics features, when using a relatively large image dataset (i.e., 3000 images in this study). This new observation supports the importance of building large and diverse image datasets in developing CAD schemes based on deep learning technologies. In addition, compared to our own previous studies that used other deep learning models, including an AlexNet [30] and a VGG-16 [29], we also observed that ResNet50 yields a higher accuracy in breast lesion classification, which supports conclusions previously reported by other researchers [23,24].

Second, after observing that the CAD scheme using radiomics features yields a lower classification performance, we conducted additional studies to analyze the contribution of using different types of radiomics features. Specifically, we divided radiomics features into three subgroups namely, (1) lesion morphology (i.e., shape) and density heterogeneity features, (2) wavelet-generated frequency domain features, and (3) texture pattern distribution features. We then applied the same PCA to create an optimal feature vector from the features in each subgroup and trained and tested the SVM model using the same 10-fold cross-validation method. We observed that performance of the three SVM models optimized using subgroups of radiomic features was lower than using the initial radiomics feature pool. The classification accuracy values for ACCs = 65.68 ± 3.02, 64.39 ± 3.14, 61.94 ± 3.42 for using three subgroups of features, respectively. However, combining all features, ACC significantly increased to 71.23 ± 2.44 (*p* > 0.01), which indicates different types or subgroups of radiomics features contain complementary discriminatory information that can be fused together to help to improve CAD performance. As a result, other types of radiomics features should also be explored in future studies.

**Figure 4:**
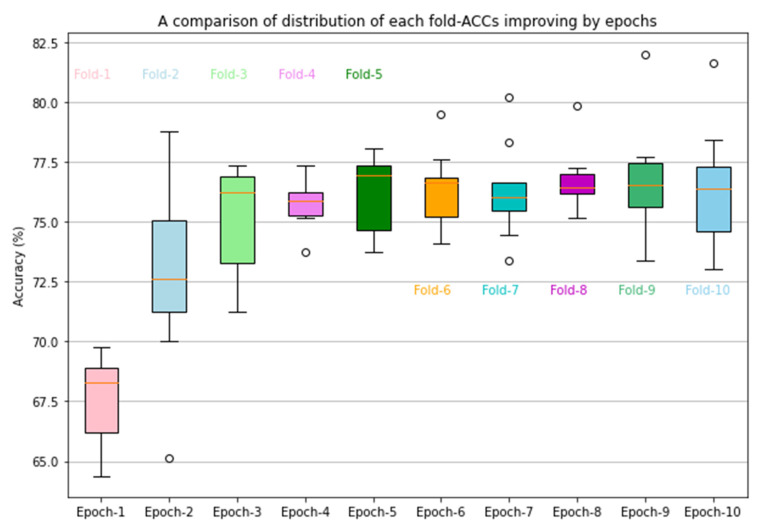
Illustration of classification accuracy and inter-fold variations in 10-fold cross validation of the CAD scheme implemented using a transfer learning ResNet50 model. Circles represent outliers observed in data analysis.

Third, a CAD scheme implemented with a deep transfer learning ResNet50 (Model-II) yields a higher lesion classification performance (as shown in Table 2). We believe that the significant classification performance improvement in comparison to Model-I is achieved by retraining or finetuning a transfer learning model to update the weights of all the layers in the network. The results demonstrate that initializing the deep learning framework with weights from pre-trained ImageNet and customizing for a binary classification task (i.e., classifying between malignant and benign breast lesions in this study) works well. This step of careful customization and training all network layers for certain epochs is essential for optimally applying the deep transfer learning network to learn the parameters used in the CAD schemes of medical images. In addition, we further analyzed the performance of Model-II in a 10-fold cross-validation. Figure 4 shows the classification accuracy (ACC) of Model-II in 10 folds. Inter-fold variation is observed, particularly, fold one has a significantly lower accuracy. The observation indicates the importance of conducting a valid statistical data analysis method (i.e., using cross-validation or a bootstrapping method) to minimize the potential bias in data partitions and to test the robustness of the deep learning models.

Fourth, in this study, we also built and tested four fusion models (Model-III.1 to Model-III.4) to detect potential performance improvement by combining Model-I and Model-II generated classification scores. In model-III.1 and Model-III.2, we used a new SVM approach and the weighted averaging methods to combine classification scores of Model-I (*S*_1_) and Model-II (*S*_2_). The results show that the classification performance metrics are very similar to Model-II, which indicates that both Model-I using radiomics features and Model-II using deep transfer learning generated automated features converge toward classification scores with a high correlation. It also supports that applying our deep transfer learning method to finetune all weights used in the ResNet50 model using mammograms is effective to characterize the lesion information difference between malignant and benign lesions. Additionally, a negative effect on performance was observed when selecting either the minimum or maximum classification score from Model-I and Model-II to serve as the final classification score of new models (Model-III.3 and Model-III.4).

Despite the above encouraging and unique observations, we also recognize some limitations in our study. First, even though we used a wide range of radiomic features (morphology, density heterogeneity, texture patterns, and wavelets-generated features) for Model-I, more radiomics features can be computed from mammograms and analyzed [11]. In addition, besides PCA, other feature dimensionality reduction methods (i.e., a locality preserving projection algorithm [31] and a random projection algorithm [9]) need to be investigated to build optimal feature vectors. Second, although an adaptive multi-layer topographic region growing algorithm is a simple and relatively robust lesion segmentation algorithm, minor manual correction is needed in a small fraction (<5%) of study cases in this large image dataset. In the future study, we will investigate the feasibility of applying deep learning-based lesion segmentation methods such as those we have investigated and used in other types of image segmentation tasks [32]. Third, we use only the standard method to finetune the ResNet50 model to conduct deep transfer learning. We need to further investigate and compare other methods, including optimal image pre-processing technologies [33] to better finetune ResNet50 or other deep learning models in the future. Fourth, we tested only four simple fusion methods to combine the classification scores of two CAD models, which is different from a more comprehensive fusion method that directly fuses radiomics features and automated features to build a new multi-feature fusion SVM model, as reported by another recent study [29]. Thus, in the future, we will try to investigate and to test more effective fusion methods after identifying more clinically relevant radiomics features, and we will try to improve the performance of radiomics feature-based machine learning models.

## 5. Conclusions

In this paper, we present a unique study that develops and tests two CAD schemes of digital mammograms to classify between malignant and benign breast lesions using two popular and advanced approaches based on radiomics and deep transfer learning concepts and technologies. Two CAD schemes or machine learning models were trained and tested, using a relatively large and diverse image dataset of 3000 images and a 10-fold cross-validation method. The study results demonstrate that although a deep transfer learning model-based CAD scheme is widely considered “a black-box” type model with a high degree of difficulty for human users to understand its learning or decision-making logic or reasoning, the automated features generated by the deep transfer learning model (i.e., ResNet50) can provide more highly discriminatory information or power than the traditional handcrafted radiomics features. More comprehensive analysis covering both radiomics and deep learning architectures needs to be further investigated to validate these observations in future studies.

## Figures and Tables

**Table 1:** Distribution of breast lesions depicting on CC and MLO view of left and right FFDM images.

Image View	Malignant Lesions	Benign Lesions	Total Lesions
Left–CC	362	368	730
Right–CC	376	409	785
Left–MLO	371	361	732
Right–MLO	387	366	753

**Table 2:** Summary and comparison of the computed areas under ROC curves (AUC) and overall classification accuracy (ACC) along with the standard deviations (STD) after applying an operation threshold (T = 0.5) to the classification scores generated by six models tested in this study.

Model (Output Score)	Feature Description	AUC ± STD	ACC (%) ± STD
Model-I (*S*_1_)	PCA-generated feature vector	0.77 ± 0.02	71.23 ± 2.44
Model-II (*S*_2_)	Transfer learning classification of ResNet50	0.85 ± 0.02	77.31 ± 2.65
Model-III.1 (*S*_3.1_)	SVM *(S*_1_, *S*_2_)	0.85 ± 0.01	77.42 ± 2.47
Model-III.2 (*S*_3.2_)	*W*_1_ × *S*_1_ + *W*_2_ × *S*_2_	0.85 ± 0.01	77.31 ± 2.83
Model-III.3 (*S*_3.3_)	Min (*S*_1_, *S*_2_)	0.83 ± 0.02	73.35 ± 2.17
Model-III.4 (*S*_3.4_)	Max (*S*_1_, *S*_2_)	0.85 ± 0.02	74.07 ± 2.24

## Data Availability

For the detailed information of image data availability for research purpose, please contact the corresponding authors.
